# The risk of upper gastrointestinal bleeding in patients treated with hemodialysis: a population-based cohort study

**DOI:** 10.1186/1471-2369-14-15

**Published:** 2013-01-16

**Authors:** Chien-Chun Kuo, Hsin-Wei Kuo, I-Ming Lee, Chien-Te Lee, Chun-Yuh Yang

**Affiliations:** 1 Division of Nephrology, Department of Internal Medicine, Kaohsiung Chang-Gung Memorial Hospital and Chang-Gung University College of Medicine, Kaohsiung, Taiwan; 2 Division of Nephrology, Department of Internal Medicine, Yuan’s General Hospital, Kaohsiung, Taiwan; 3 Department of Public Health, Kaohsiung Medical University, 100 Shih-Chuan 1st RD, Kaohsiung 80708, Taiwan; 4 Division of Environmental Health and Occupational Medicine, National Health Research Institute, Miaoli, Taiwan

**Keywords:** Upper gastrointestinal bleeding, End-stage renal disease, Cohort study

## Abstract

**Background:**

There are no prior studies that have estimated the risk of upper gastrointestinal bleeding (UGIB) among the dialysis population relative to the general population. The aim of this study was to examine the risk of UGIB among end-stage renal disease (ESRD) patients during a 6-year period following their initiation of hemodialysis (HD) therapy in Taiwan- a country with the highest incidence of ESRD in the world, using general population as an external comparison group.

**Methods:**

Data were obtained from the Taiwan National health Insurance Research Database. In total, 796 patients who were beginning HD between 1999 and 2003 were recruited as the study cohort and 3,184 patients matched for age and sex were included as comparison cohort. Multivariate Cox proportional hazard regression models were used to adjust for confounding and to compare the 6-year UGIB-free survival rate between these two cohorts.

**Results:**

The incidence rate of UGIB (42.01 per 1000 person-year) was significantly higher in the HD cohort than in the control cohort (27.39 per 1000 person-years). After adjusting for potential confounders, the adjusted hazard ratios for UGIB during the 6-year follow-up periods for HD patients was 1.27 (95% CI=1.03-1.57) compared to patients in the comparison cohort.

**Conclusions:**

We conclude that HD patients were at an increased risk for UGIB compared with the general population.

## Background

The increasing number of patients with end-stage renal disease (ESRD) requiring dialysis therapy is becoming a worldwide public health problem and puts a substantial burden on health care resources. Taiwan is also confronting this severe challenge [1]. The incidence and prevalence of ESRD in Taiwan were not only the highest of some 30 countries reported by USRDS, but were 2–4 times higher than most European countries [2]. There have been 2.6- and 3.7-fold increases in incidence and prevalence during the past decade in Taiwan, respectively [1]. The financial burden of dialysis has exceeded the expenditure on all cancers combined, and reducing the number of people with ESRD constitutes a national health priority in Taiwan [3].

Studies suggest that acute gastrointestinal hemorrhage, especially upper gastrointestinal bleeding (UGIB), is the most frequent bleeding complication of acute renal failure (ARF), even though the reported incidence ranges widely [4]. Reports also suggest that the prevalence or incidence of UGIB in patients with ESRD may be greater than that in the general population [5-10]. It has been estimated that UGIB accounts for 3 to 7% of all deaths among ESRD patients [5]. However, some studies found that patients with chronic renal failure are not at risk for developing chronic peptic ulcers [11,12]. Most of the previous studies were conducted in western countries [5,6,8,10-12] and were done before the era of extensive use of pharmacological prophylaxis of upper gastrointestinal stress lesions in critically ill patients [4].

To date, there are limited data regarding the occurrence of UGIB in dialysis patients. Most of the above-mentioned epidemiologic studies suffer from methodological limitations such as a relatively small size, case series without controls or not taking potential confounders into considerations in the regression model. With these methodological issues in mind, we conducted a cohort study using general population as an external comparison group to determine the risk of UGIB among ESRD patients during a 6-year period following their initiation of hemodialysis (HD) therapy in Taiwan- a country with the highest incidence of ESRD in the world. To our knowledge, this is the first population-based cohort study to investigate the relationship between HD and the risk of UGIB.

## Methods

### Data source

The National Health Insurance (NHI) program, which provides compulsory universal health insurance, was implemented in Taiwan on March 1, 1995. Under the NHI, 98% of the island’s population receives all forms of health care services including outpatient services, inpatient care, Chinese medicine, dental care, childbirth, physical therapy, preventive health care, home care, and rehabilitation for chronic mental illness. In cooperation with the Bureau of NHI, the National Health Research Institute (NHRI) of Taiwan randomly sampled a representative database of 1,000,000 subjects from the entire NHI enrollees by means of a systematic sampling method for research purposes. There were no statistically significant differences in age, gender, and healthcare costs between the sample group and all enrollees, as reported by the NHRI. This dataset (from January 1996 to December 2009) includes all claim data for these 1,000,000 subjects, offers a good opportunity to examine the risk of UGIB occurring among patients with ESRD. These databases have previously been used for epidemiological research, and information on prescription use, diagnoses, and hospitalizations has been shown to be of high quality [1,13-15].

Because the identification numbers of all individuals in the NHRI databases were encrypted to protect the privacy of the individuals, this study was exempt from full review by the Kaohsiung Medical University Institution Review Board.

### Study cohorts

From this database, we selected all patients who were beginning chronic HD or peritoneal dialysis between January 1, 1999 and December 31, 2003 and who have survived more than 90 days of renal replacement therapy for ESRD (n=1046) [16]. We excluded individuals younger than 18 years of age because they were not at appreciable risk of UGIB and in order to limit the study subjects to an adult population (n=9). ESRD patients are defined as those who had catastrophic illness registration cards for ESRD (ICD-9-CM code 585) and started renal replacement therapy. In Taiwan, patients who reached ESRD with the need for long-term renal replacement therapy can apply for catastrophic illness registration cards given by the Bureau of Health Insurance. These patients do not need to pay copayments when they seek health care for renal disease. A detailed description of this cohort is published elsewhere [17]. We excluded subjects with any type of UGIB diagnosed before or within 90 days of their index ambulatory care visit (the date of a patient’s initiating dialysis) (n=169). In addition, we also excluded those subjects who received a renal transplant (n=3) or peritoneal dialysis (n=57). A total of 808 incident HD patients were identified between January 1, 1999 and December 31, 2003.

The comparison cohort were selected from the remaining patients in the database. We first excluded patients who had been diagnosed with chronic kidney disease (ICD-9 CM codes 250.4^*^, 274.1^*^, 283.11, 403.^*^1, 404.^*^2, 404.^*^3, 440.1, 442.1, 447.3, 572.4, 580–588, 642.1^*^, 646.2^*^) [1] during the period 1996–2009 and those younger than 18 years of age. For each study cohort patient, 4 reference subjects were identified randomly and matched for gender, age (birth of year), and the year of index ambulatory care visit. For the comparison cohort, the index ambulatory care visit was their first ambulatory care visit occurring in the index year. In addition, we excluded patients who had been diagnosed with UGIB before or within 90 days of their index ambulatory care visits. Of the 808 incident HD cases ascertained, no controls could be found for 12 of the cases. A total of 3,184 subjects served as a comparison cohort group.

### Incidence of UGIB

Patients were considered to have experienced UGIB if a hospital discharge diagnosis indicating UGIB, as defined by any one of the gastric, duodenal, peptic, and gastrojejunal bleeds were reported (ICD-9-CM codes 531.0X, 531.2X, 531.4X, 531.6X, 532.00, 532.2X, 532.4X, 532.6X, 533.0X, 533.2X, 533.4X, 533.6X, 534.0X, 534.2X, 534.4X, 534.6X, 535.X1). Gastrointestinal bleed, unspecified, was not used to identify UGIB because it was felt to be too nonspecific with regard to location of bleeding.

### Potential confounders

For all individuals in both cohorts, we obtained data on potential confounders which are documented risk factors for UGIB, including hypertension (ICD-9-CM, codes 401–405), diabetes (ICD-9-CM, code 250), hyperlipidemia (ICD-9-CM, code 272), coronary heart disease (ICD-9-CM, codes 410–414), chronic liver disease and cirrhosis (ICD-9-CM, code 571), and Helicobacter pylori (HP) eradication, recorded during 12 months before the index ambulatory care visit. In addition, we also obtained prescription data for medications for aspirin, non-steroidal antiinflammatory drugs (NSAIDs), H_2_ receptor antagonists (H_2_RA), and proton pump inhibitor (PPI) use, recorded between the index date to the date of UGIB hospitalization, death or the end of the study. We collected the date of prescription, the daily dose, and the number of days supplied. The defined daily doses (DDDs) recommended by the WHO were used to quantify the usage of medications [18].

### Statistics

For comparisons of proportions between the study and comparison cohorts, the chi-square test was used. The mean doses of medication use (aspirin, NSAIDs, H_2_RA, and PPI) were calculated and compared between the study and comparison cohorts using paired t test; because of the skew distribution of these medication use data, medication use data were log-transformed to improve normality. For the analysis, the subjects were categorized into one of the three exposure categorizes: nonusers (subjects with no prescription at any time between the index date and the date of UGIB hospitalization, death or the end of the study), and users of doses equal to or below the mean, and users of doses above the mean based on the distribution of use among controls. Each patient was individually tracked for a 6-year period starting from the index ambulatory care visit to identify whether the patient had experienced UGIB during the follow-up period. The person-years of follow-up were calculated for each patient from the date of the index ambulatory care visit to the date of UGIB hospitalization, death or the end of the study (a 6-year follow-up period), whichever occurred first. Incidence rates were calculated by dividing the number of UGIB hospitalizations by the total person-years of follow-up. Kaplan-Meier curves and log-rank tests were used to explore the difference in the risk of developing UGIB between the 2 cohorts. A multivariate frailty Cox proportional hazards regression model, which incorporates an unmeasured “random” effect (the frailty) in the hazard function, was used to estimate the hazard ratios (HR) and 95% confidence intervals (CI) adjusted for the above-mentioned potential confounders [19]. The assumption of proportional hazards was assessed by including an interaction term between time and exposure (HD patients vs patients in the comparison cohort) in the model, and the proportional assumption was satisfied. Analyses were performed using the SAS statistical package (version 9.2, SAS Institute Inc., Cary, NC, USA). All statistical tests were two-sided. Values of p < 0.05 were considered statistically significant.

## Results

There were 796 patients in our HD cohort that were compared with 3,184 selected matched controls. Table 1 presents the distribution of demographic characteristics and selected medical conditions of the study subjects. Of the 3,980 patients sampled, the mean age was 56.72 years (SD=13.74), with means of 56.72 (SD=13.75) and 56.72 (SD=13.74) for HD patients and comparison patients, respectively. HD patients had a significant higher rate of hypertension, diabetes, hyperlipidemia, coronary heart disease, and liver cirrhosis. HD patients were also significantly more likely to have PPI use.

**Table 1:** Basic characteristics for hemodialysis patients versus the comparison patients

**Variable**	**HD patients**		**Comparison patients**		**p value**
	**n=796**		**n=3184**		
	**n**	**%**	**n**	**%**	
Gender					
Male	365	45.85	1460	45.85	-
Female	431	54.15	1724	54.15	
Age (mean±SD)	56.72±13.75		56.72±13.74		-
< 50	241	30.28	964	30.28	0.99
50-64	296	37.19	1184	37.19	
65-74	200	25.13	798	25.06	
> 74	59	7.41	238	7.47	
Hypertension					
Yes	566	71.11	1032	32.41	< 0.001
No	230	28.89	2152	67.59	
Diabetes					
Yes	288	36.18	395	12.41	< 0.001
No	508	63.82	2789	87.59	
Coronary heart disease					
Yes	162	20.35	372	11.68	< 0.001
No	634	79.65	2812	88.32	
Hyperlipidemia					
Yes	145	18.22	446	14.01	0.003
No	651	81.78	2738	85.99	
Liver cirrhosis					
Yes	20	2.51	33	1.04	0.001
No	776	97.49	3151	98.96	
HP eradication					
Yes	54	6.78	201	6.31	0.627
No	776	97.49	3151	98.96	
Aspirin use (gm/person-year)	11.28±35.76		13.05±47.29		0.247
NSAIDs use (DDDs/person-year)	14.72±29.54		41.99±75.14		< 0.001
PPI use (DDDs/person-year)	16.15±184.80		3.02±16.39		< 0.001
H_2_RAs use (DDDs/person-year)	6.97±21.30		5.60±18.44		0.490

Table 2 shows the distribution of UGIB during the 6-year follow-up periods for these two cohorts. Of the total 3,980 sampled patients, 647 patients (16.26%) developed UGIB throughout the study periods, 176 (22.11% of the HD patients) from the study cohort (with an incidence rate of 42.01 per 1,000 person-years) and 471 (14.79%) from the comparison cohort (with an incidence rate of 27.39 per 1,000 person-years). Gastric ulcers were found to be the most common source of bleeding (16.23 per 1000 person-years), while bleeding resulting from a gastrojejunal ulcer was least frequent (0.48 per 1000 person-years) among HD patients.

**Table 2:** Crude hazard ratios of UGIB in hemodialysis cohort compared to patients in comparison cohort during the 6-year follow-up period

	**HD patients**	**Comparison patients**
	**(n=796)**	**(n=3184)**
No. of UGIB	176	471
Person time (years)	4189.06	17196.73
Incidence^a^	42.01	27.39
Crude HR (95% CI)	1.50 (1.25-1.80)	1.00
No. of gastric ulcer	68	144
Person time (years)	4189.06	17196.73
Incidence^a^	16.23	8.37
Crude HR (95% CI)	1.86 (1.37-2.51)	1.00
No. of duodenal ulcer	26	86
Person time (years)	4189.06	17196.73
Incidence^a^	6.21	5.00
Crude HR (95% CI)	1.21 (0.77-1.90)	1.00
No. of peptic ulcer	50	160
Person time (years)	4189.06	17196.73
Incidence^a^	11.94	9.30
Crude HR (95% CI)	1.24 (0.89-1.72)	1.00
No. of gastrojejunal ulcer	2	10
Person time (years)	4189.06	17196.73
Incidence^a^	0.48	0.58
Crude HR (95% CI)	0.94 (0.20-4.44)	1.00
No. of gastritis/duodenitis with bleeding	30	71
Person time (years)	4189.06	17196.73
Incidence^a^	7.16	4.13
Crude HR (95% CI)	1.74 (1.12-2.70)	1.00

^a^ per 1000 person-years.

**Figure 1:**
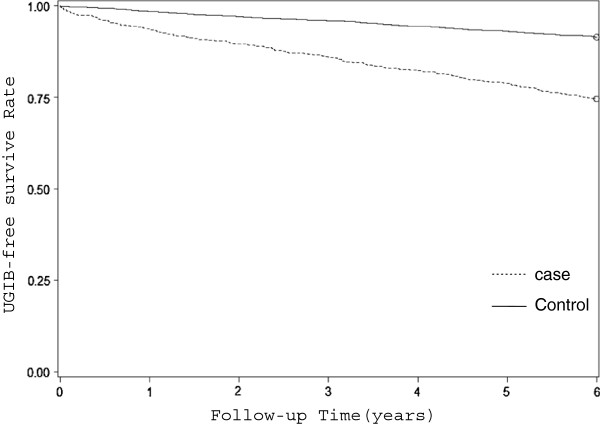
UGIB-free survive rates for HD patients and patients in the comparison cohort.

After adjusting for age, gender, hypertension, diabetes, hyperlipidemia, coronary heart disease, liver cirrhosis, the use of NSAIDs, and PPI, Cox multivariate hazard regression analysis showed that the hazard of UGIB during the 6-year follow-up periods was 1.27 times greater (95% CI=1.03-1.57) for patients treated with hemodialysis than for the comparison cohort (Table 3; Figure 1).

**Table 3:** Adjusted hazard ratio (HRs) for UGIB among the sampled patients identified by Cox regression analysis

**Variable**	**Adjusted HR (95% CI)**^**a**^
Patients receiving HD	1.27 (1.03-1.57)
Hypertension	1.13 (0.91-1.39)
Diabetes	1.09 (0.86-1.39)
Coronary heart disease disease	1.14 (0.88-1.49)
Hyperlipidemia	1.14 (0.88-1.46)
Liver cirrhosis	1.14 (0.59-2.20)
Medication use	
NSAIDs (per 1 DDD increase)	1.00 (0.99-1.00)
PPI (per 1 DDD increase)	1.01 (1.00-1.01)

^a^ Adjusted for all covariates listed.

## Discussion

The only study estimating the incidence of UGIB in dialysis patients is performed in the United States, which reported an incidence rate of 22.77 per 1000 person-years [10]. The incidence of UGIB was 42.01 per 1000 person-years in the present study. The reason for a higher incidence rate of UGIB in Taiwanese HD patients are not clear. It may be that renal transplant recipients and peritoneal dialysis patients were excluded in the present study and that renal transplant recipients and peritoneal dialysis patients had a lower risk of UGIB compared to HD patients [10]. However, the possibility that the incidence rates of UGIB in Taiwanese population are higher than that of the United States and that characteristics of HD patients are substantially different from that in the United States can not be excluded. Further study is needed to clarify the reason.

There are no prior studies that have estimated the risk of UGIB among the dialysis population relative to the general population. Previous study has only used dialysis patients as a cohort to identify patient characteristics associated with the incident UGIB without seeking a comparison group [10]. Because of the uniqueness of the exposure (hemodialysis), special exposure cohort (HD patients) provides a statistically efficient way of studying this factor, which would be rare in general cohort. Special exposure cohort (dialysis patients), however, require an external comparison group. To the best of our knowledge, this study is among the first matched cohort study to examine the risk of UGIB among HD patients using general population as an external comparison group (with a age- and gender-matched comparison group). In this population-based cohort study, after adjusting for potential confounders, we found that HD patients were 1.27 times more likely than the general population to experience UGIB during the 6-year follow-up period. Our findings support previous studies that found chronic renal failure can be a UGIB risk factor.

The major strength of our study is the use of a computerized database, which is population based and is highly representative and allows a clear observation of the temporal relationship between HD and UGIB. Because we included a national sample of ESRD patients initiating HD therapy between January 1, 1999 and December 31, 2003, and because the control subjects in this study were selected from a simple random sampling of insured general population, we can rule out the possibility of selection bias. Moreover, the large sample size affords considerable statistical power for detecting real difference between the two cohorts.

Several limitations of the present study should be noted. First, diagnoses of ESRD, UGIB, or any other comorbid medical conditions and prescription information rely on administrative claims data may be less accurate than those obtained according to standardized criteria and misclassification is possible. However, from previous studies, the NHIRD is of acceptable quality to provide reasonable estimations for epidemiological data [13-15]. Moreover, regular chart-review and cross-checking mechanisms conducted by Taiwan’s NHI Bureau do facilitate the accuracy of coding [20]. Second, although we adjusted for several potential confounders in the statistical analysis, a number of possible confounding variables, including smoking [21], alcohol use [22], physical inactivity [21,23], and Helicobacter pylori infection [24], which might be associated with UGIB development were not included in our database. These unmeasured risk factors might have biased results if they were differentially associated with case versus comparison cohort. Third, we were not able to contact the patients directly about their use of medications because of anonymization of their identification number. Using pharmacy records representing dispensing data rather than usage data might have introduced an overestimation of medication use. Fourth, the case cohort includes only HD patients. It has been hypothesized that intermittent anticoagulation may place HD patients at greater risk for UGIB. If this were the case, one would anticipate that peritoneal dialysis and renal transplantation would be associated with a lower risk for UGIB compared to HD [10]. Therefore, the results of this study may not be generalizable to patients receiving peritoneal dialysis or renal transplantation.

## Conclusion

In summary, our study results provide evidence that HD patients are at an increased risk for UGIB compared with general population. Therefore, prevention of UGIB is becoming an important issue in the maintenance of good quality of life for HD patients.

## Competing interests

All authors have no conflict of interests; This paper is neither the entire paper nor any part of its content has been published or accepted elsewhere.

## Authors’ contributions

KCC did the statistical analysis and wrote the manuscript. KHW, LIM and LCT provided essential insight into the interpretation of the results. YCY contributed to study design and interpretation of the data. All authors read and approved the final manuscript.

## Pre-publication history

The pre-publication history for this paper can be accessed here:

http://www.biomedcentral.com/1471-2369/14/15/prepub
